# Expression of the Multiple Sclerosis-Associated MHC Class II Allele HLA-DRB1*1501 Is Regulated by Vitamin D

**DOI:** 10.1371/journal.pgen.1000369

**Published:** 2009-02-06

**Authors:** Sreeram V. Ramagopalan, Narelle J. Maugeri, Lahiru Handunnetthi, Matthew R. Lincoln, Sarah-Michelle Orton, David A. Dyment, Gabriele C. DeLuca, Blanca M. Herrera, Michael J. Chao, A. Dessa Sadovnick, George C. Ebers, Julian C. Knight

**Affiliations:** 1 Wellcome Trust Centre for Human Genetics, University of Oxford, Oxford, United Kingdom; 2 Department of Clinical Neurology, University of Oxford, John Radcliffe Hospital, Oxford, United Kingdom; 3 Department of Medical Genetics, Division of Neurology, University of British Columbia, UBC Hospital, Vancouver, British Columbia, Canada; 4 Faculty of Medicine, Division of Neurology, University of British Columbia, UBC Hospital, Vancouver, British Columbia, Canada; The Jackson Laboratory, United States of America

## Abstract

Multiple sclerosis (MS) is a complex trait in which allelic variation in the MHC class II region exerts the single strongest effect on genetic risk. Epidemiological data in MS provide strong evidence that environmental factors act at a population level to influence the unusual geographical distribution of this disease. Growing evidence implicates sunlight or vitamin D as a key environmental factor in aetiology. We hypothesised that this environmental candidate might interact with inherited factors and sought responsive regulatory elements in the MHC class II region. Sequence analysis localised a single MHC vitamin D response element (VDRE) to the promoter region of *HLA-DRB1*. Sequencing of this promoter in greater than 1,000 chromosomes from *HLA-DRB1* homozygotes showed absolute conservation of this putative VDRE on HLA-DRB1*15 haplotypes. In contrast, there was striking variation among non–MS-associated haplotypes. Electrophoretic mobility shift assays showed specific recruitment of vitamin D receptor to the VDRE in the HLA-DRB1*15 promoter, confirmed by chromatin immunoprecipitation experiments using lymphoblastoid cells homozygous for HLA-DRB1*15. Transient transfection using a luciferase reporter assay showed a functional role for this VDRE. B cells transiently transfected with the HLA-DRB1*15 gene promoter showed increased expression on stimulation with 1,25-dihydroxyvitamin D3 (*P* = 0.002) that was lost both on deletion of the VDRE or with the homologous “VDRE” sequence found in non–MS-associated *HLA-DRB1* haplotypes. Flow cytometric analysis showed a specific increase in the cell surface expression of *HLA-DRB1* upon addition of vitamin D only in HLA-DRB1*15 bearing lymphoblastoid cells. This study further implicates vitamin D as a strong environmental candidate in MS by demonstrating direct functional interaction with the major locus determining genetic susceptibility. These findings support a connection between the main epidemiological and genetic features of this disease with major practical implications for studies of disease mechanism and prevention.

## Author Summary

Multiple Sclerosis (MS) is a complex neurological disease with a strong genetic component. The Major Histocompatibility Complex (MHC) on chromosome 6 exerts the strongest genetic effect on disease risk. A region at or near the *HLA-DRB1* locus in the MHC influences the risk of MS. *HLA-DRB1* has over 400 different alleles. The dominant haplotype of Northern Europe, marked by the presence of DRB1*1501, increases risk of MS by 3-fold. The environment also plays a key role in MS. The most striking illustration of this is the geographical distribution of the disease in populations matched for ethnicity. This has led to the proposal that sunshine, and in particular, vitamin D, is an environmental factor influencing the risk of MS. Circumstantial evidence supporting this comes from studies showing the involvement of vitamin D in immune and nervous system function. The current investigation sought to uncover any relationship between vitamin D and *HLA-DRB1*. It was found that vitamin D specifically interacts with HLA-DRB1*1501 to influence its expression. This study therefore provides more direct support for the already strong epidemiological evidence implicating sunlight and vitamin D in the determination of MS risk, and implies that vitamin D supplementation at critical time periods may be key to disease prevention.

## Introduction

Multiple sclerosis (MS) is a common inflammatory disease of the central nervous system characterized by myelin loss, axonal pathology, and progressive neurological dysfunction [1]. The aetiology of MS is unknown, however it is clear that genetic and environmental components are important [1],[2].

The only genetic association with MS in Northern Europeans had been with extended MHC haplotypes, especially those containing HLA-DRB1*1501 [3]. The interleukin 7 receptor (*IL7RA*), interleukin 2 receptor (*IL2RA*), ecotropic viral integration site 5 (*EVI5*) and kinesin family member 1B (*KIF1B*) genes have recently been shown to be additional MS susceptibility loci [4],[5],[6],[7]. The largest of these, *KIF1B*, has a relatively small effect size (odds ratio (OR) = 1.3). The MHC (OR = 5.4) is the key susceptibility locus in MS and other susceptibility genes identified to date appear to contribute little to overall risk [3].

The principal MHC class II haplotype that increases MS risk in individuals of Northern European descent is HLA- DQB1*0602-DQA1*0102 -DRB1*1501-DRB5*0101 [8], although other *HLA-DRB1* haplotypes have important influences on risk by epistatic interactions [9],[10],[11],[12]. Intense linkage disequilibrium within the MHC has frustrated attempts at fine mapping and no precise susceptibility locus has been identified [9],[13].

Twin studies have established that monozygotic (MZ) twin concordance is significantly greater than for dizygotics (DZ). In the study by Willer and colleagues concordance was 25.3% and 5.4% respectively [14]. The observation that most MZ twin pairs are discordant for MS suggests environmental, stochastic factors or both but the most striking illustration of the importance of the environment in MS susceptibility is the 5-fold difference in MS risk between Tasmania and Queensland [15]. In the Northern Hemisphere, MS prevalence shows a north-south gradient, mirrored by a south-north gradient in the southern hemisphere (reviewed by [16]).

In accordance with the disease geography, sunlight, specifically through its role in generating active vitamin D, has been proposed as a key environmental factor for the disease [17]. Circumstantial evidence to support this comes from studies showing that MS patients are deficient in vitamin D [18] and that dietary vitamin intake reduces disease risk [19]. Additionally, a pooled analysis of over 40,000 patients from Canada, Great Britain, Denmark, and Sweden showed that fewer people with MS were born in November and more in May [20], highlighting a risk factor that varies seasonally. Vitamin D is primarily known for its critical role in calcium homeostasis, however recent evidence has highlighted many actions on immune and central nervous system development and function [21]. These have contributed to the notion that this is how vitamin D affects MS risk, although direct links have not yet been identified.

Vitamin D is a secosteroid hormone synthesized in the skin or ingested in the diet. Intake from dietary sources accounts for a much smaller proportion of total vitamin D, mainly owing to its rarity in foods [22],[23]. During exposure to sunlight, ultraviolet B (UVB) radiation (290–315 nm) is responsible for photolyzing 7-dehydrocholesterol, the precursor of vitamin D3, to previtamin D3 which, in turn, rapidly spontaneously isomerizes to vitamin D3 [22],[23]. Vitamin D3 is biologically inert and requires hydroxylation in the liver to 25-hydroxyvitamin D3 (25(OH)D). Once formed, this major circulating form of vitamin D3 is further hydroxylated in the kidney to its active form, 1,25-dihydroxyvitamin D3 (1,25(OH)_2_D), by 25-hydroxyvitamin D-1α-hydroxylase (1-OHase). Recently it has been recognized that most tissues in the body (including the brain, thymus and cells of the immune system) also possess the 1-OHase enzyme. Thus numerous tissues in the body have the capacity to locally produce 1,25(OH)_2_D [22],[23].

Most biological effects of 1,25-dihydroxyvitamin D3 or calcitriol, are mediated by the vitamin D receptor (VDR). This receptor is a member of the steroid receptor super-family and influences the rate of transcription of vitamin D responsive genes by acting as a ligand activated transcription factor that binds to vitamin D response elements (VDREs) in gene promoters [21]. Early studies had provided evidence for an effect of vitamin D on HLA gene expression [24],[25], although no specific mechanism has been characterised. Here we examined the hypothesis of a direct interaction between vitamin D and MS associated MHC class II genes. Genetic variation characteristic of the most significant risk haplotypes for MS, those bearing HLA-DRB1*15, includes a functional vitamin D response element (VDRE) in the proximal promoter region of *HLA-DRB1*. This provides a mechanism linking the major environmental and genetic risk factors for MS.

## Results

### *In Silico* Identification of Putative Vitamin D Response Elements

**Figure 1:**
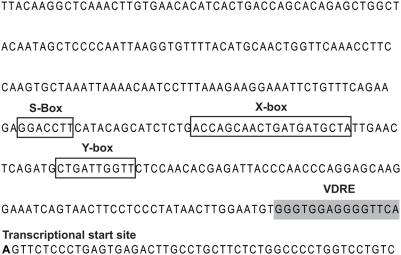
HLA-DRB1 promoter. Sequence shown is that for HLA-DRB1*15. Important regulatory elements (S, X and Y Boxes) are highlighted.

Using the sequence for the HLA-DRB1*15 haplotype carried by the homozygous lymphoblastoid cell line PGF we scanned *in silico* for VDREs using Jaspar [26] with a profile score threshold of 80%. We analysed the entire genomic sequence of the *HLA-DRB1*, *HLA-DQA1* and *HLA-DQB1* genes as well as 5 kb upstream of the transcriptional start sites of these genes to include promoter regions. VDREs exhibit a multitude of sequence variations, providing a spectrum of binding affinities for VDR, thus enabling these elements to respond to differing concentrations of VDR/1,25(OH)_2_D [22]. The analysis revealed only one potential VDRE located in the proximal promoter region immediately 5′ to the transcriptional start site of *HLA-DRB1* (Figure 1). *IL2RA* and *IL7RA* were also searched *in silico* for potential VDR binding sequences; no putative VDREs were found.

### Sequencing of the *HLA-DRB1* Promoter in MS Patients and Controls

The occurrence and conservation of the putative VDRE element identified in the PGF sequence was examined in individuals with the HLA-DRB1*15 MS risk allele. The *HLA-DRB1* promoter was resequenced in 322 HLA-DRB1*15 homozygous individuals, both MS affected and unaffected. An additional 168 individuals homozygous for other *HLA-DRB1* alleles were also sequenced. The putative VDRE was present on all HLA-DRB1*15 bearing haplotypes with no variants found which disrupted the VDRE consensus sequence. In contrast, a number of nucleotide changes were found within the 15 base pairs of the VDRE on all non-HLA-DRB1*15 haplotypes. For example, nearly all (98% of 57 sequenced individuals) of HLA-DRB1*04, HLA-DRB1*07 and HLA-DRB1*09 haplotypes, all of which are non-MS associated alleles in the Canadian population [10], carried the sequence GGGTGGAGAGGGGTCA. This sequence was predicted to function less effectively as a VDRE than the one on HLA-DRB1*15 bearing haplotypes according to Jaspar [26]. The modestly MS associated haplotype, HLA-DRB1*17, differed from HLA-DRB1*15 at the VDRE in 50% of the individuals sequenced.

### *In Vitro* Binding of VDR to the HLA-DRB1*15 VDRE

**Figure 2:**
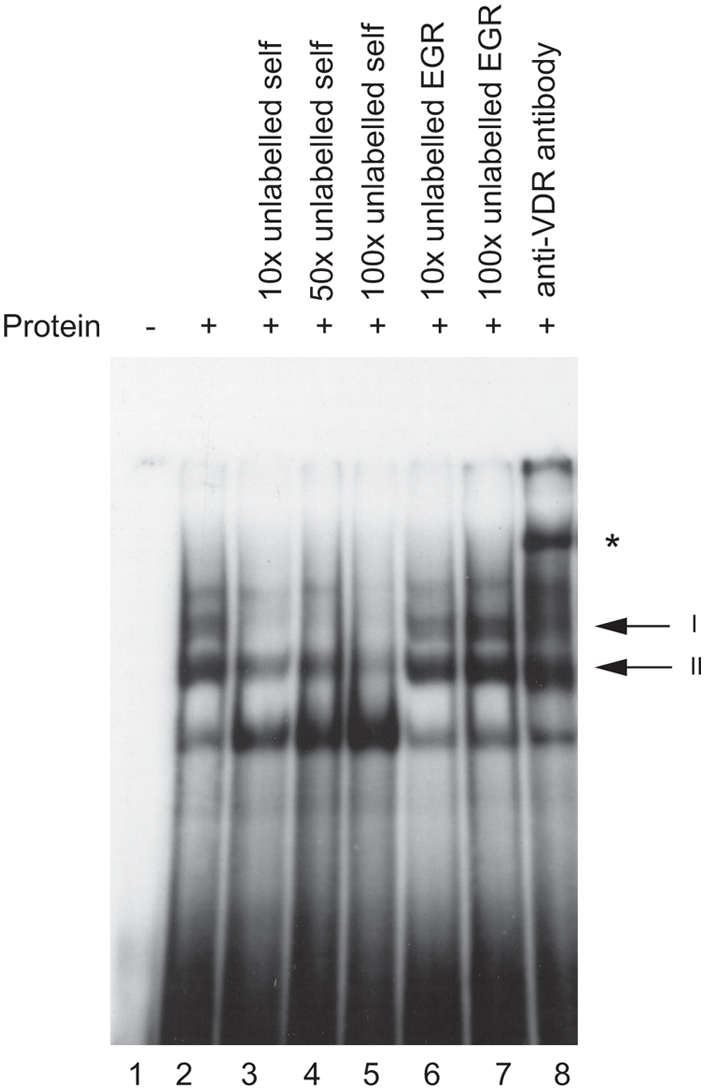
In vitro binding of VDR protein to the HLA-DRB1*15 VDRE. Electrophoretic mobility shift assay showing binding of recombinant VDR and retinoic acid receptor beta (RXR) to radiolabelled oligoduplex probe corresponding to the VDRE in the proximal *HLA-DRB1* promoter region for the HLA-DRB*15 haplotype. Two specific complexes are indicated, denoted I and II, together with a supershifted complex shown by an * symbol in the presence of antibody to VDR.

The putative VDRE in the *HLA-DRB1* promoter was investigated for ability to bind the vitamin D receptor *in vitro* using an electrophoretic mobility shift assay (EMSA). Upon addition of recombinant VDR and retinoic acid receptor beta (RXR, a co-regulator of VDR binding and transactivation [22]) to a radiolabelled probe spanning the putative VDRE in the *HLA-DRB1* promoter, two protein-DNA complexes on EMSA were observed (Figure 2, lane 2). Both complexes were specifically competed with 10 to 100-fold molar excess of unlabelled VDRE probe (Figure 2, lanes 3–5), while 10 to 100 fold molar excess of an unrelated probe containing an early growth response (EGR) factor binding site had no effect (Figure 2, lanes 6–7). Finally, addition of a polyclonal antibody directed against VDR specifically retarded complex I, resulting in a supershift of the upper complex (Figure 2, lane 8). This data showed the putative VDRE in the *HLA-DRB1* promoter corresponding to the HLA-DRB1*15 haplotype could bind recombinant VDR/RXR with high specificity *in vitro*. When probes corresponding to the HLA-DRB1*04/07/09 variant VDRE were used, significantly lower affinity binding was found (*data not shown*).

### Evidence *Ex Vivo* of VDR Binding to the VDRE Found on the HLA-DRB1*15 Haplotype

**Figure 3:**
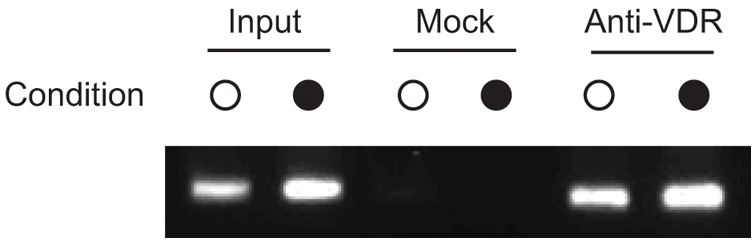
VDR is recruited to HLA-DRB1*15 VDRE in PGF cells. Chromatin immunoprecipitation experiment using PGF cells either unstimulated (○) or after stimulation with 1,25-dihydroxyvitamin D3 (•). Input controls are shown (lanes 1 and 2), mock antibody immunoprecipitated controls (lanes 3 and 4) and VDR primary antibody immunoprecipitated DNA (lanes 5 and 6).

Whether or not the VDR is recruited to the VDRE in the *HLA-DRB1* gene promoter was examined *ex vivo*. Chromatin immunoprecipitation (ChIP) experiments were performed using lymphoblastoid cells bearing the HLA-DRB1*15 haplotype (the PGF cell line) which were either unstimulated or stimulated for 24 hours with 1,25-dihydroxyvitamin D3 and then cross-linked in the presence of formaldehyde. Immunoprecipitation was performed using antibodies against VDR. The VDR bound DNA fragments were then recovered after reversal of protein-DNA crosslinking and analysed by PCR using primers specific for the *HLA-DRB1* promoter. A representative agarose gel is shown in Figure 3. This revealed clear evidence of binding by VDR to the *HLA-DRB1* promoter when compared to input chromatin and mock antibody controls for cells with the HLA-DRB1*15 haplotype, complementing the *in vitro* data from the EMSA experiments.

### Transient Transfection

**Figure 4:**
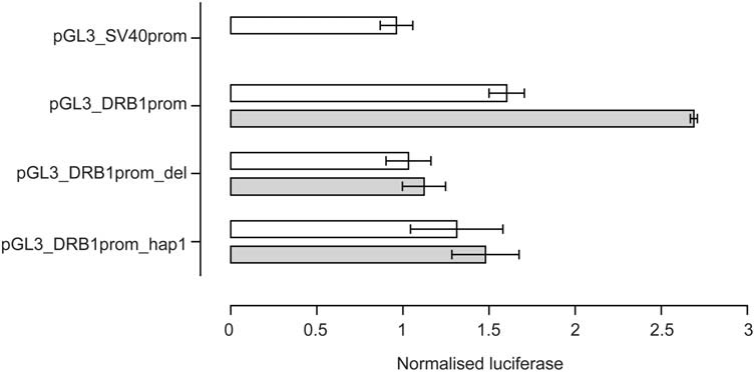
Reporter gene analysis of DRB1 promoter VDRE. Raji B cells were transiently transfected with pGL3 luciferase constructs as indicated together with pRL_TK to normalise luciferase activity. Open bars indicate resting cells, grey shaded bars results following stimulation of transfected cells with 1,25-dihydroxyvitamin D3. Mean+/−SD of three independent transient transfection experiments are shown, each performed in quadruplicate.

The VDRE was then investigated to see if it modulated levels of gene expression *in vitro*. Reporter gene constructs were engineered in which −181 to +53 of the *HLA-DRB1* gene sequence was placed upstream of a pGL3 luciferase reporter. pGL3_DRB1prom had the complete −181 to +53 sequence, pGL3_DRB1prom_hap1 had the same sequence as pGL3_DRB1prom but the VDRE replaced with the HLA-DRB1*04/07/09 VDRE and pGL3_DRB1prom_del had the 15 base pair VDRE sequence specifically deleted. These constructs were then transiently transfected into Raji B cells. A renilla luciferase reporter construct driven by the thymidine kinase promoter (pRL_TK) was co-transfected to normalise luciferase activity. pGL3_DRB1prom had significantly higher basal reporter gene activity than pGL3_DRB1prom_del (*P* = 0.03 on paired *t*-test, two tailed). After stimulation with 1,25-dihydroxyvitamin D3, there was a significant 1.6 fold increase in luciferase activity with pGL3_DRB1prom (*P* = 0.002), but no significant change with pGL3_DRB1prom_del (*P* = 0.12), nor pGL3_DRB1prom_hap1 (*P* = 0.58) (Figure 4).

### Flow Cytometry

To investigate any effect of vitamin D on the cell surface expression of *HLA-DRB1*, the HLA-DRB1*15 homozygous lymphoblastoid cell line PGF and the HLA-DRB1*07 homozygous lymphoblastoid DBB cell line were stained with anti-*HLA-DRB1* antibody. PGF cells constitutively expressed *HLA-DRB1* at higher levels then DBB (average geometric mean fluorescence intensity (MFI) PGF = 97.1, DBB = 42.8, *P* = 0.0002). Upon addition of 1,25-dihydroxyvitamin D3, there was a 1.3 fold increase in the expression of *HLA-DRB1* in PGF cells (*P* = 0.031 on paired *t*-test, two tailed) but no significant difference in the expression of *HLA-DRB1* in DBB cells (*P* = 0.10).

## Discussion

While the role of the environment is clearly important in determining MS risk, the relevant underlying mechanism(s) have remained elusive and there has been no experimental support for a direct environment-gene interaction. Although differences in Epstein-Barr virus infection are seen when MS patients are compared to controls, extensive searches for specific viral infections have failed to confirm direct involvement. [2]. Where appropriate data is available, the amount of winter sunlight parallels the range of MS prevalence, and high sunlight exposure is associated with low disease prevalence [2]. The effects of migration between high and low risk geographic regions have been examined in several populations (e.g.UK immigrants to South Africa, or Asian and Caribbean immigrants to the UK). These studies show that MS risk is influenced by the migrant's country of origin [27]. Despite the limits of small sample sizes, a ‘critical age’ has been hypothesized: immigrants who migrate before adolescence acquire the risk of their new country, while those who migrate after retain the risk of their home country. Dietary difference for vitamin D intake (oily fish consumption) plausibly explains the striking exception to MS latitudinal risk in Norway [2]. As familial aggregation is genetically determined [28], environmental factors thus appear to be operative at a broad population level, perhaps acting at a young age [27] and/or during gestation [20]. A good candidate for an environmental factor that influences MS disease risk is vitamin D.

We approached the candidacy of vitamin D by searching first for vitamin D response elements within the MHC class II region. Specifically we investigated the major candidate genes in the disease associated locus, *HLA-DRB1*, *HLA-DQA1* and *HLA-DQB1* and identified a consensus binding site for VDR next to the *HLA-DRB1* gene. This was the only VDRE we found and strikingly it shows haplotype-specific differences, being highly conserved in the major MS associated haplotype HLA-DRB1*15 dominant in Northern European populations, but not conserved among non-MS associated haplotypes. This was itself circumstantial evidence supporting a vitamin D role in the functional characteristics of this haplotype. The identified VDRE lies close to the highly conserved MHC class II specific regulatory SXY module. This module comprises S, X and Y regulatory elements important for constitutive, and indirectly for IFN-γ-induced, expression of HLA class II genes co-ordinated by the MHC class II transactivator *MHC2TA* [29]. The VDRE was highly conserved on HLA-DRB1*15 haplotypes (no mutations on over 600 chromosomes) suggesting a selective pressure to maintain this response element for the HLA-DRB1*15 allele. Variants were found to some extent on all other non HLA-DRB1*15 haplotypes. The results may additionally/alternatively reflect the ancestral origin of the HLA-DRB1*15 (DR51) haplotype [30] which displays the strongest linkage disequilibrium among the MHC class II haplotypes [31]. We note the association between this haplotype and MS risk is characteristic of Northern European populations, the ones most vulnerable to vitamin D deficiency [2].

EMSA experiments using recombinant proteins demonstrated that *in vitro* VDR can bind specifically to the putative VDRE in the proximal *HLA-DRB1* promoter found on the HLA-DRB1*15 haplotype. ChIP data showed specific enrichment of the region spanning the VDRE in VDR immunoprecipitated samples relative to input and mock antibody controls, demonstrating that the vitamin D receptor was recruited to this haplotype in this *ex vivo* model system. Finally, transient transfection and flow cytometric assays established that the VDRE present in the *HLA-DRB1* promoter can influence gene expression and imparts 1,25-dihydroxyvitamin D3 sensitivity to HLA-DRB1*15. The variant VDRE present on other, non-MS associated *HLA-DRB1* haplotypes was not responsive to 1,25-dihydroxyvitamin D3.

A T cell repertoire with millions of specificities provides surveillance against a multitude of foreign pathogens [32]. An inherent danger in recognizing so many foreign proteins is the potential to respond to self-proteins. To circumvent this problem T cells are scrutinised for self-reactivity as they mature in the thymus with deletion of those posing the greatest threat (central deletion) [32]. One constraint on central deletion is the requirement for the relevant autoantigen to be present in the thymus. Whether or not these are expressed as proteins at levels sufficient to induce T cell deletion is not clear. Given the results of this study, variable expression of *HLA-DRB1* could affect central deletion of autoreactive T cells. It is plausible that a lack of vitamin D *in utero* or early childhood can affect the expression of *HLA-DRB1* in the thymus, and impacting on central deletion. For MS, in HLA-DRB1*15 bearing individuals, a lack of vitamin D during early life could allow auto reactive T cells to escape thymic deletion and thus increase autoimmune disease risk. Indeed it has been shown that antigen presentation in the thymus of VDR knock-out mice is impaired [33]. However the mechanism for a HLA- vitamin D interaction remains unclear as is the timing and tissue in which such interactions might occur. A major selective pressure on skin pigmentation is thought to have been vitamin D deficiency with progressively lighter skin pigmentation at increasing distance from the equator related to variation in intensity of ultraviolet radiation with latitude [34]. The presence of a VDRE specific to HLA-DRB1*15- bearing haplotypes, present at high allele frequencies among Northern Europeans, suggests a possible role for vitamin D in selection at this locus. The intriguing possibility that vitamin D responsiveness rather than any antigen-specificity determines the increased MS risk of the HLA-DRB1*15 haplotype warrants consideration and can be tested in the infrequent haplotypes bearing the VDRE on other non-HLA-DRB1*15 haplotypes.

In summary, we have identified and functionally characterised a vitamin D response element (VDRE) in the *HLA-DRB1* promoter region. These studies imply direct interactions between *HLA-DRB1*, the main susceptibility locus for MS, and vitamin D, a strong candidate for mediating the environmental effect. This study provides more direct support for the already strong epidemiological evidence implicating sunlight and vitamin D in the determination of MS risk. Given that a high frequency of vitamin D insufficiency in the general population has been observed [35], our data support the case for supplementation during critical time periods to reduce the prevalence of this devastating disease.

## Materials and Methods

### Subjects, HLA-DRB1 Genotyping, Sequencing

All participants in the study were ascertained through the ongoing Canadian Collaborative Project on the Genetic Susceptibility to MS (CCPGSMS) [36]. Subject ascertainment, genotyping and sequencing has been previously described [9],[10],[37].

### Ethical Statement

Each participating clinic in the CCPGSMS obtained ethical approval from the relevant institutional review board, and the entire project was reviewed and approved by the University of British Columbia and the University of Western Ontario.

### EMSA

EMSAs were performed as previously described [38]. The VDRE probe comprised of the annealed sense and antisense strands of the nucleotide sequence agctGTGGGTGGAGGGGTTCATAG, the EGR probe agctAAATCCCCGCCCCCGCGATGGA and the VDRE variant probe agctGTGGGTGGAGAGGGGTCATAG. Full length recombinant purified VDR and recombinant purified RXR beta were purchased from Invitrogen, and polyclonal VDR antibody from Affinity Bioreagents. Radioactivity was quantitated with the Packard Cyclone phosphorimager, and analyzed with Optiquant (Perkin Elmer Life Sciences). Values were compared using the Chi square test.

### Chromatin Immunoprecipitation

The lymphoblastoid cell line PGF was cultured in RPMI-1640 medium supplemented with 10% fetal bovine serum, 0.2 mM L-glutamine at 37°C in 5% humidified CO_2_. 60×10^6^ cells were harvested unstimulated or after stimulation with 0.1 uM calcitriol (Sigma). Cells were crosslinked using a 1% formaldehyde buffer for 15 minutes at room temperature, quenched with glycine and chromatin prepared as previously described [39]. Chromatin was sheared by sonication in the presence of 212–300 microns glass beads (Sigma) at 4°C using a double step microtip attached to a Branson 450 Sonifier with coupler (Branson) in 30 second bursts (six pulses at 40%) with the samples cooled on ice for 1 minute between pulses. Sonicated chromatin was then processed and subject to immunoprecipitation as previously described [39] using magnetic ‘Dynabeads M-280’ (Dynal) precoated with anti rabbit IgG to which the primary antibody VDR was bound (Affinity Bioreagents). We followed the buffer used for immunoprecipitation and subsequent washes as described [40]. Following reversal of crosslinks, RNase A and Proteinase K digestion, DNA was extracted using phenol-chloroform and amplified by PCR with separation on a 2.0% agarose gel. The primers used for PCR were: forward- GCAACTGGTTCAAACCTTCC and reverse- GTCCCCAGACAAAGCCAGT. Cycling conditions were: 95°C for 10 minutes; a touchdown of 14 cycles (95°C for 30 seconds; 61°C with −0.5°C per cycle, for 30 seconds; 72°C for 30 seconds); 35 cycles of 95°C for 30 seconds, 53.5°C for 30 seconds, 72°C for 30 seconds; 72°C for 7 minutes.

### Cell Transfection and Luciferase Reporter Gene Assay

The plasmids were constructed by inserting the promoter region (−181 to +53) of the human *HLA-DRB1* gene (pGL3_DRB1prom with the VDRE sequence (chr6:32,665,500–32,665,760), pGL3_DRB1prom_del with the VDRE sequence deleted (chr6:32,665,500–32,665,559 combined with chr6:32,665,575–32,665,760)) into the pGL3 reporter plasmid. Two independent plasmid preparations were used in transient transfection experiments for each construct.

Raji B cells were cultured in RPMI-1640 medium supplemented with 10% fetal bovine serum, 0.2 mM L-glutamine at 37°C in 5% humidified CO_2_. Lipofectamine-LTX and PLUS reagent (Invitrogen) were used for transient transfection of expression constructs, following the manufacturer's protocol. pRL_TK was co-transfected to normalize for transfection efficiency. When indicated, cells were stimulated with 0.1 uM calcitriol (Sigma) for 24 hours. Cells were harvested after 24 hours and lysed in 500 ul of 1× lysis buffer (Promega) and analyzed using the Dual-Luciferase reporter assay kit (Promega) and a Turner luminometer model 20 (Promega) following the manufacturer's protocol. Paired *t*-tests were used to compare expression values. Each transfection was carried out 12 times in total.

### Flow Cytometry

The lymphoblastoid cell lines PGF (International Histocompatibility Workshop number IHW09318) and DBB (IHW09052) were cultured in RPMI-1640 medium supplemented with 10% fetal bovine serum, 0.2 mM L-glutamine at 37°C in 5% humidified CO_2_. 1×10^6^ cells were harvested unstimulated or 24 hours after stimulation with 0.1 uM calcitriol (Sigma) in three biological replicates. Cells were stained with either a FITC conjugated monoclonal anti-human HLA-DR antibody (Sigma, F1902) or a FITC conjugated isotype control antibody (Sigma, F6522) for 30 minutes at room temp, then washed with 2% BSA in PBS and re-suspended in 1 mL of 2% paraformaldehyde. Cells were analysed using CyAn flow cytometer (Dako).
